# Psychometric properties of the Arabic version of the Fertility Quality of Life (FertiQoL) questionnaire tested on infertile couples in Jordan

**DOI:** 10.1186/s12905-023-02437-6

**Published:** 2023-05-25

**Authors:** Salwa Al Obeisat, Audai Hayajneh, Issa Hweidi, Sanaa Abujilban, Lina Mrayan, Rana Alfar, Abdulqadir Nashwan, Lama I. Hweidi

**Affiliations:** 1 Faculty of Nursing\Maternal-Child Health Nursing Department, Jordan University of Science and Technology, Irbid, 22110 Jordan; 2 Faculty of Nursing\Adult Health Nursing Department, Jordan University of Science and Technology, Irbid, 22110 Jordan; 3 Department of Maternal, Child and Family Health Nursing, Faculty of Nursing, The Hashemite University, Zarqa, 13115 Jordan; 4 Nursing for Education & Practice Development, Hazm Mebaireek General Hospital (HMGH), Hamad Medical Corporation (HMC), Doha, Qatar; 5 Faculty of Medicine, Jordan University of Science & Technology, Irbid, 22110 Jordan

**Keywords:** Psychometric properties, Arabic, Fertility quality of life, Jordanian, Infertile couples

## Abstract

**Background:**

The Fertility Quality of Life (FertiQoL) questionnaire assesses the quality of life of people with fertility problems. The present study aimed to assess the psychometric properties of the Arabic version of the FertiQoL in infertile couples in Jordan.

**Methods:**

This study used a cross-sectional design among 212 participants with infertility problems. An exploratory factor analysis (EFA) and a confirmatory factor analysis (CFA) were conducted to investigate the underlying structure of the novel Arabic version of the FertiQoL tool.

**Results:**

The Cronbach’s alpha values for the FertiQoL core domain, the FertiQoL treatment domain, and the total FertiQoL scale were 0.93, 0.74, and 0.92, respectively. The EFA indicated a two-domain model, with the first factor having 24 items and measuring "Core QoL". The second factor has 10 items and measures "Treatment QoL" in the context of infertility. The EFA and the CFA supported a two-factor model whereby the two factors explained 48% of the shared covariance between the analyzed quality of life indicators. The indices of goodness-of-fit of the model showed acceptable fit as follows: the chi-squared test (χ2) = 7.943, the comparative fit index (CFI) = 0.999, the root mean square error of approximation (RMSEA) = 0.001, and the Tucker-Lewis index (TLI) = 0.989.

**Conclusion:**

The study's findings demonstrated the reliability and validity of the Arabic version of the FertiQoL for assessing the quality of life of infertile couples or those in Jordan with no pregnancy or childlessness.

## Introduction

Infertility is a global health concern that impacts both women and men and significantly impacts their lives. Worldwide, 10–15% of couples of childbearing age experience infertility [1, 2]. According to the World Health Organization (WHO), approximately 48 million couples and 186 million people worldwide are infertile [3]. Of those infertile couples, 19.2 million have primary infertility and 29.3 million have secondary infertility [4].

Infertile couples from different cultures around the world can experience social suffering and stigma due to their lack of pregnancy and childlessness [5]. Infertility can lead to various distressing outcomes that may be related to gender, culture, fertility history, and infertility diagnosis [6]. How people deal with infertility depends on their values, social norms, and religious backgrounds [6, 7].

Infertility affects all aspects of couples' lives and poses significant challenges for those who wish to father children [2], as well as having an impact on the health and well-being of individuals, couples, and society as a whole [8]. Infertility can lead to adverse social, physical, and psychological effects, including depression, anxiety, marital intimidation, helplessness, economic hardship, abandonment, social isolation, physical violence, and social stigma backgrounds [6, 7].

Numerous studies have shown how infertility and its treatments negatively affect infertile couples’ quality of life. (QOL) [9, 10]. In addition, it has been shown that QOL is lower in infertile women compared to infertile men [9], and evidence suggests that women are at a higher risk than men of developing emotional problems [11]. Infertile women who are particularly at risk of reduced QOL are those who are older, of lower educational level, or unemployed [12]. As compared to employed infertile men, unemployed infertile men are more likely to have significantly lower mean quality of life scores in the physical health and social relations domains [13]. Similar to infertile women, infertile men experience significant levels of depression and anxiety, and thus to achieve more effective interventions and outcomes for infertile couples, health professionals must include men in their assessments [14].

The inability to get pregnant becomes an issue that stigmatizes and emotionally impacts infertile couples [15, 16]. In Arab cultures, getting married and having children is one of life's greatest accomplishments, and when something like infertility becomes a problem, the accompanying stress and stigma can have significant impacts on infertile couples’ lives [17]. Many married women are subjected to social pressure to have children, and many women worry that if they do not, their husbands will remarry or divorce them. Infertile individuals often consider separation or divorce as the best solution to not being able to give their partner a child [18]. These aforementioned stressors can result in a lower quality of life and self-esteem in infertile couples [19, 20].

In addition, couples who have failed infertility treatment experience less social support, more sexual dissatisfaction, and an increased risk of suicide [21, 22]. However, infertile couples with higher levels of education, higher income, and shorter infertility periods are more likely than their infertile counterparts to be more satisfied with their marital relationships’ problems [11, 12, 23].

The Fertility Quality of Life [FertiQoL] questionnaire was developed by an international group of experts in English to assess the quality of life of infertile couples, and its preliminary psychometric properties have been evaluated by Boivin and colleagues [24]. The questionnaire has been translated into 26 different languages, including Arabic, and used among various populations from different cultures. However, few studies have investigated the psychometric properties of the FertiQoL questionnaire [24]. The Arabic version of the FertiQoL questionnaire is an internationally developed and validated questionnaire to measure the infertility-specific quality of life. The questionnaire consists of two general items including: 1) How would you rate your health? and 2) Are you satisfied with your quality of life?) and two modules to measure the quality of life (the core module with 24 questions and the optional treatment module with 10 questions [17]. The questionnaire is available on the FertiQoL website at http://sites.cardiff.ac.uk/fertiqol/. To the best of the researchers' knowledge, the psychometric properties of the Arabic version of the FertiQoL questionnaire have never been evaluated., therefore the purpose of the current study was to evaluate the psychometric properties of the Arabic version of the FertiQoL questionnaire in a sample of infertile couples in Jordan.

## Methods

### Research design

A cross-sectional descriptive design was employed to validate the FertiQoL questionnaire's Arabic version in a Jordanian sample of infertile couples.

### Setting and sampling

This study was conducted in the infertility treatment units of three selected hospitals in the northern region of Jordan. These hospitals serve approximately three million people and provide various medical and counseling services, including infertility and fertilization treatment and counseling services. A non-probability, convenience sampling technique was utilized because of its cost-effectiveness and time-efficiency to get access to data from infertile subjects who are difficult to approach within the Jordanian culture which regards infertility as a sensitive issue that can’t be disclosed easily [25]. A sample of 180 participants was required based on Thorndike’s rule of thumb [26]. An additional 30% of the required sample size was added to account for any possible dropouts or non-response (54 participants) ending with a final sample was 234 participants. Of these 22 participants withdrew from the study and were therefore excluded. The final sample consisted of 212 participants (106 couples) who consented to participate and met the eligibility criteria. These criteria were Jordanian, at least 18 years old, literate in Arabic, married for at least 12 months, and seeking fertility treatment. Non-Jordanians and couples married for less than a year were excluded.

### Instrument

The Fertility Quality of Life (FertiQoL) questionnaire is an internationally developed and validated self-report questionnaire designed to assess the quality of life for those who are having fertility problems [24]. It consists of two general items:1) How would you rate your health? and 2) Are you satisfied with your quality of life?) and two modules (core module 24-item and optional treatment module 10-item) [17]. The core module items assess the effect of infertility on all life aspects and are categorized into four domains, namely the mind–body (six items), relational (six items), social (six items), and emotional (six items) domains. The mind–body domain evaluates how infertility affects one's physical well-being, cognition, and behavior, the relational domain on the partnership, and the social domain on social issues like social support, social inclusion, and expectations. The optional treatment module items assess the environment and tolerability of fertility treatment and are categorized into the environment domain (six items) and the tolerability domain (four items). Psychometric analyses have reported high Cronbach’s alphas throughout these domains (range 0.72–0.92) [24].

The FertiQoL items are scored on a five-point scale ranging from 0 to 4. The scale yields six subscale scores and three total scores ranging from 0 to 100, with higher scores indicating a higher quality of life. The FertiQoL scoring did not include the two general items aimed at evaluating overall physical health and satisfaction with the quality of life. The total FertiQoL score indicates the average quality of life across the core and treatment domains. The scoring process consisted of three steps: 1) reverse the items; 2) compute raw scores by adding all items from the subscale or total scale. For the Core-FertiQoL domain, we added all 24 items, and for the Treatment-FertiQoL domain, we added all 10 items. For the total FertiQoL, we added all core and treatment items (34 items); 3) multiply the relevant raw score by 25/k, where k is the number of items in the subscale.

### Ethical considerations

Ethical approval (Ref#22/95/2016) to carry out the study was obtained from the Institutional Review Board (IRB) at Jordan University of Science and Technology (JUST) and the selected hospitals. All eligible couples who agreed to participate signed a consent form which included details about the purpose of the study, the data collection procedure, the time required to complete the questionnaire, and the risks and benefits of participating in the study. In addition, the consent form included a clear statement that highlighted those participants had the right to withdraw at any time without penalty. Furthermore, the participants were informed that their responses to the survey would be kept confidential and that a code would be assigned to each questionnaire, whereby names or any personal information would not be used.

### Data collection

After approval to conduct the study was obtained from the JUST IRB and the selected hospitals, the principal investigator scheduled a meeting with the director of each infertility unit to explain the purpose and data collection procedure. The director of each infertility unit then provided the principal investigator with a list of the infertile couples who attended the infertility clinic. The principal investigator approached all couples on these lists, who briefly explained the study aims and procedures and invited them to participate in the study. Those who agreed were screened for eligibility to participate in the study based on the aforementioned criteria.

Eligible participants who consented to participate then signed the informed consent forms. The consent form included a clear explanation of the purpose of the study and the risks and benefits of participating. After that, the questionnaires were handed out to the participants to fill out, and they took about 15–20 min to fill out the questionnaire. During the data collection period, the principal investigator was available at a close distance to answer any questions or provide clarifications. Both male and female partners were asked to complete the questionnaire separately Each completed questionnaire was assigned a separate code (starting from 001). Data collection took place from June to August 2016.

## Statistical analysis

Continuous variables were described using means and standard deviations, and categorically measured variables were described using frequencies and percentages. Histograms and the K-S statistical test of normality were used to assess the statistical normality assumption of the continuous variables. The reliability analysis was tested using Cronbach's alpha and Item-scale correlations analysis. Items with corrected item-total correlations < 0.30 were considered for deletion. The items of the questionnaire were all recoded to that the greater their score will denote the greater their quality of life.

To assess the factorial validity and structure of the Fert-QoL questionnaire, the exploratory factor analysis (EFA), the principal components analysis (PCA), and the parallel analysis were used to assess the existing number of dimensions within the scale and the un-dimensionality of the 34-indicators of Fert-QoL. The confirmatory factor analysis (CFA) was then applied to the six subscales of the Fert-QoL to assess the presence of a first-order latent factor based on the EFA, that is, the presence of two latent factors as proposed by the authors. The goodness of fit of the proposed model was assessed using the chi-squared goodness-of-fit index, the Tucker-Lewis Index (TLI), the comparative fit index (CFI), and the root mean squared error approximate (RMSEA) index. The SPSS IBM statistical analysis program Version 25, SPSS AMOS program version 20, and the stand-alone FACTOR program were used for the statistical data analysis. The level of statistical significance was set at 0.05.

## Results

The total number of infertile respondents in the study was 212 (106 male and 106 female respondents). The mean and standard deviation of the respondents’ ages were 33.16 ± 6.77, with 40.6% (*n* = 84) of the respondents aged between 20–30 years, 46.7% (*n* = 99) aged between 31–40 years, and 13.7% (*n* = 29) aged 41 years or over. More than half of the participants (56%; *n* = 119) lived in urban areas and 38.2% (*n* = 88) in rural areas. Most study participants (84.4%; *n* = 179) lived with their nuclear families, while 15.6% (*n* = 33) lived with their extended families. More than half of the study participants (53.8%; *n* = 114) had a monthly income of 500 JOD or less, 16% (*n* = 34) between 501–800 JOD, and 30.2% (*n* = 64) over 800 JOD. More than half of the participants (52.0%; *n* = 104) had a bachelor's degree, 37.5% (*n* = 89) had a high school diploma, and 10.5% (*n* = 22) had one Postgraduate Degree. About two-thirds of the study participants (69.8%; *n* = 148) had no health insurance. Among the couples in the study sample, the mean duration of marriage in years was 7.58 years (SD = 4.92 years). Most participants (67.5%; *n* = 148) had arranged marriages, while 32.5% (*n* = 69) had romantic marriages. The predominant type of infertility was primary infertility with a total of 154 participants (72.6%) while 58 participants (27.4%) had secondary infertility (Table 1).

**Table 1:** Descriptive analysis of the infertility patients’ sociodemographic characteristics. *N* = 212

	**Frequency**	**Percentage**
**Gender**		
Female	106	50.0
Male	106	50.0
**Age(years), mean (SD)**		**33.16 (6.77)**
**Age group**		
20–30 years	84	40.6
31–40 years	99	46.7
41–50 years or higher	29	13.7
**Educational Level**		
High school or less	80	37.5
University Degree	104	52.0
Master’s degree and more	22	10.5
**Place of Residence**		
Urban	119	56.1
Rural	93	43.9
**Type of Family**		
Nuclear	179	84.4
Extended	33	15.6
**Households’ monthly income (JOD), mean (SD)**		**747.10 (643.70)**
**Income levels**		
< = 500 JOD	114	53.8
501–800 JOD	34	16.0
= > 801 JOD	64	30.2
**Medical Insurance**		
No	148	69.8
Yes	64	30.2
**Duration of marriage (years), mean (SD)**		**7.58 (4.92)**
**Type of marriage**		
Arranged marriage	143	67.5
Romantic marriage	69	32.5
**Type of infertility**		
Primary	154	72.6
Secondary	58	27.4

### Reliability

The internal consistency Cronbach's alpha test was applied to the 34-indicator-long FertiQoL questionnaire and its subscales, along with the post-hoc item-total correlations and scale if item deletion reliability analysis. The analysis findings showed that the 24 items of the fertility core quality of life subscale were read and answered by people equally reliably, with a Cronbach's alpha of 0.93. The fertility treatment quality of life subscale (10 items) was also measured reliably, with a Cronbach’s alpha of 0.74, and the 34 indicators analyzed together showed satisfactory results, with a Cronbach's alpha of 0.92.

Considering the six subscales of the overall FertiQoL, the analysis showed that four items had low corrected item-total correlations (< 0.30) and that their deletion from their subscales could enhance the internal consistency results for the subscales. These items were 1) Ferti-QoL4R Do you feel able to cope with your fertility problems? 2) Ferti-QoL15R Have fertility problems strengthened your commitment to your partner? 3) Ferti-QoL14R Do you feel your family can understand what you are going through? and 4) Ferti-QoLT2R Are the fertility medical services you would like available to you? (Table 2).

**Table 2:** Reliability analysis of the measured scales

	**Number of items**	**Cronbach's alpha**
**Fertility core quality of life**	26	0.93
**Fertility treatment Quality of life**	10	0.74
**Overall Fertility Quality of life**	36	0.92

### Validity

 As presented in Table 3, the EFA findings indicated that the 34 indicators of the FertiQoL questionnaire were suitable to be analyzed based on satisfactory Kaplan-Meyer-Olkins index of sampling adequacy (KMO = 0.886), and a significant Bartlett’s test of Sphericity (χ2(630) = 4197, *p* < 0.001). These latter tests suggested the absence of unwanted collinearity between the QoL indicators and the presence of an invertible and factorable correlation matrix between those indicators.

**Table 3:** First Order item-factor loadings for the fertility quality of life indicators

	Item#			**First Order Item-Factor Loadings**	
Item#4 excluded		**Emotional QoL**			
5 items	C8	fertQol8 Do you experience grief and/or feelings of loss about not being able to have a child (or more children)?		0.883	
	C7	fertQol7 Do your fertility problems cause feelings of jealousy and resentment?		0.864	
	C16	*fertQol16 Do you feel sad and depressed about your fertility problems?*		0.864	
	C9	fertQol9 Do you fluctuate between hope and despair because of fertility problems?		0.818	
	C23	fertQol23 Do your fertility problems make you angry?		0.742	
		**Mind–Body QoL**			
6 items	C3	fertQol3 Do you feel drained or worn out because of fertility problems?		0.865	
	C2	fertQol2r Do you think you cannot move ahead with other life goals and plans because of fertility problems?		0.828	
	C18	fertQol18 Are you bothered by fatigue because of fertility problems?		0.826	
	C1	fertQol1 Are your attention and concentration impaired by thoughts of infertility?		0.808	
	C24	fertQol24 Do you feel pain and physical discomfort because of your fertility problems?		0.759	
	C12	fertQol12 Do your fertility problems interfere with your day-to-day work or obligations?		0.630	
Item15r excluded		**Relational QoL**			
5 items	C19	fertQol19 Have fertility problems had a negative impact on your relationship with your partner?		0.853	
	C20	fertQol20, Do you find it difficult to talk to your partner about your feelings related to infertility?		0.778	
	C6	fertQol6 Are you satisfied with your sexual relationship even though you have fertility problems?		0.695	
	C21	fertQol21R Are you content with your relationship even though you have fertility problems?		0.543	
	C11	fertQol11R Are you and your partner affectionate with each other even though you have fertility problems?		0.467	
Item14R removed		**Social QoL**			
5 items	C17	fertQol17 Do your fertility problems make you inferior to people with children?		0.850	
	C15	fertQol5, Are you satisfied with the support you receive from friends about your fertility problems?		0.788	
	C22	fertQol22, Do you feel social pressure on you to have (or have more) children?		0.750	
	C10	fertQol10 Are you socially isolated because of fertility problems?		0.612	
	C13	fertQol13, Do you feel uncomfortable attending social situations like holidays and celebrations because of your fertility problems?		0.578	
Item2T removed		**Environmental QoL**			
5 items	T9	fertQolT9 How would you rate the quality of information you received about medication, surgery, and/or medical treatment?		0.866	
	T8	fertQolT8 How would you rate the surgery and/or medical treatment(s) you have received?		0.848	
	T10	fertQolT10 Are you satisfied with your interactions with fertility medical staff?		0.833	
	T7	fertQolT7 Are you satisfied with the quality of services available to you to address your emotional needs?		0.723	
	T5	fertQolT5R Do you feel the fertility staff understands what you are going through?		0.462	
		**Tolerability QoL**			
4 items	T4	fertQolT4 Are you bothered by the effect of treatment on your daily or work-related activities?		0.884	
	T6	fertQolT6 Are you bothered by the physical side effects of fertility medications and treatment?		0.810	
	T1	fertQolT1 Does infertility treatment negatively affect your mood?		0.803	
	T3	fertQolT3 How complicated is dealing with the procedure and/ or administration of medication for your infertility treatment(s)?		0.677	

However, the interim parallel analysis (PA test) and the scree-plot test showed that there were only two latent factors that could be retrieved from the 34 indicators, and these two latent factors explained 48% of the shared covariance between the analyzed indicators of QoL. All the core indicators loaded saliently on the first factors, and the treatment factors loaded saliently on the second factor, except for a few problematic indicators which were excluded from the analysis.

First-order item factor loadings for the indicators comprising each of the six subscales of FertiQoL were requested by the analysis program, as shown in Table 3, to further identify problematic items The resulting interim EFA of those subscales suggested that the items (C4: Do you feel able to cope with your fertility problems? C14: Do you feel your family can understand what you are going through? C15: Have fertility problems strengthened your commitment to your partner? and T2 Are the fertility medical services you would like available to you?) had high residual error loading with their parent subscale factors. However, items (#4: Do you feel able to cope with your fertility problems? and #14: Do you feel your family can understand what you are going through?) had low (< 0.30) initial extracted variance. Item#15: Have fertility problems strengthened your commitment to your partner? and item#T2: Are the fertility medical services you would like available to you? loaded weakly to their parent factors with item-factor loading < 0.10. Therefore, these problematic items were excluded, and the factor analysis was repeated without them, as can be noted in Table 3. This resulted in indicators that had loaded saliently (> 0.45) to their parent factors.

Then total scores for each of the six subscales were calculated by adding up people's perceptions of each of the indicators, excluding the aforementioned problematic items. The subscales were then rescaled into scores between 0–100, as described in the FertiQoL scoring manual. Then, the six indicators were tested with the CFA using the SEM, as proposed by the authors of the questionnaire.

**Fig. 1:**
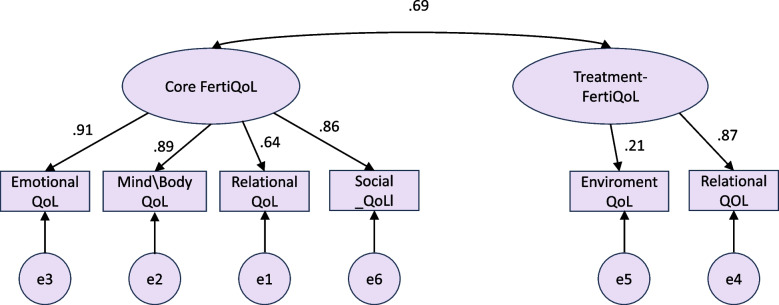
The confirmatory factor analysis for the Arabic version of FertiQol (*N* = 212)

The CFA shown in Table 4 was applied to the six subscale items of the FertiQoL, as proposed by Boivin et al. [23] via the structural equation modeling program using second order and measurement first-order models. The second-order latent factor analysis did not fit the data well, and a first-order measurement model was tested, as shown in Fig. 1 and Table 4. The chi-squared test of the goodness-of-fit test showed excellent fit between the proposed model with the covariance matrix between the six subscales, with χ2 (8) = 7.943, minimum discrepancy per degree of freedom (CMIN/DF) = 0.993, *p*-value = 0.439, TLI = 0.989, CFI = 0.999, RMSEA fit index = 0.001, 90% C. I (0.002, 0.083), and PCLOSE = 0.742. As a result, all indices indicated a good fit between the proposed model and the measured data, conforming to the author's proposed theoretical model (see Table 5).

**Table 4:**
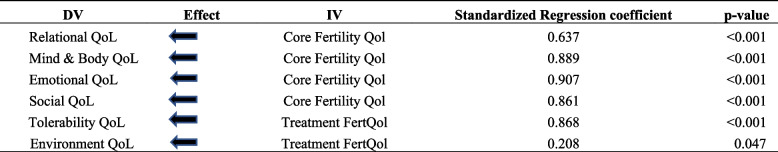
The confirmatory factor analysis (CFA) of the Ferti-Qol questionnaire first-order measurement model

*IV* Independent predictor variable/exogenous variable, *DV* Dependent/endogenous variable. Model Fit indices: CFI fit index = 0.999, TLI fit index = 0.989, RMSEA = 0.001, 90% C.I RMSEA:0.002: 0.083, PCLOSE = 0.742

**Table 5:** The goodness-of-fit indices of the model

The goodness-of-fit indices	CMIN/DF (*p*-value)	RMSEA, 90% C.I (Low, High)	χ2 (df)	TITLE	CFI
The value	0.993 (0.44)	0.001 (0.002, 0.083)	7.943 (8)	0.989	0.999

*χ2* Chi-squared test, *CMIN* Minimum discrepancy per degree of freedom, *TLI* Tucker-Lewis index, *CFI* Comparative fit index, *RMSEA* Root mean squared error approximate, *CI* Confidence interval, *df* degree of freedom

Table 5 displays the standardized regression coefficients between the subscales and their parent latent factors (core and treatment quality of life overall scales). All the indicators loaded substantively (> 0.60) to their parent latent QoL factors in standardized scales, indicating their convergent validity to their proposed latent factors. For example, there was a significant positive correlation between the relational subscale with the core fertility parent factor (beta = 0.637). However, the environmental subscale loaded with a relatively low standardized regression coefficient (Beta = 0.208, *p*-value = 0.047) on the treatment QoL upper factor. The two latent quality of life domains (core and treatment) correlated significantly and positively, *r* = 0.69, *p* < 0.001, denoting their convergent validity.

## Discussion

This is the first study to evaluate the psychometric properties of the Arabic version of the FertiQoL for use with Jordanian infertile couples. In the current study, the two main subscales of the FertiQoL had either acceptable or excellent Cronbach’s alpha values for internal consistency and reliability, ranging from 0.74 to 0.93. These excellent and acceptable values are similar to those reported by Maroufizadeh and colleagues [27], ranging from 0.64 to 0.9. Similarly, Cronbach's alpha values of over 0.70 were reported in the study by Priangga and colleagues [28]. Regarding the inter-item correlations between the items of the two main parts of the FertiQoL, the current study results showed a positive correlation between the treatment module of the QoL and the core module (emotional, mind/body, relational and social). Furthermore, the core module domains correlated positively with the tolerability domain of the treatment module. This means that the QoL of both female and male partners is affected by all domains of QoL, and similar findings have been reported for infertile couples in the Netherlands and Iran [29, 30]. In contrast, Taiwanese infertile men have higher emotional and mind/body scores than females [29–31].

In the current study, we found four items with low item-total correlations (< 0.30), as follows: fertiqol4R: Do you feel able to cope with your fertility problems?; ferti-Qol15R: Have fertility problems strengthened your commitment to your partner?; ferti-Qol14R: Do you feel your family can understand what you are going through?; and ferti-QolT2R: Are the fertility medical services you would like available to you? Those low values could be referred to as non-related infertility concepts, such as copying, commitment, and connections toward self, family, and medical services. Furthermore, Maroufizadeh and colleagues [27] recommended the removal of the two items “Q15: Have fertility problems strengthened your commitment to your partner?” and “T2: Are the fertility medical services you would like available to you?” due to their low loading values on the “relational” and “environment” domains. Further studies should be conducted to investigate the aforementioned items and their connections to the main two subscales of the FertiQoL tool.

Moreover, a previous Dutch study that aimed to assess the reliability of the FertiQoL examined the relationship between emotional distress as measured by the Hospital Anxiety and Depression Scale (HADS) and the FertiQoL questionnaire. the findings showed a negative association between quality of life and anxiety and depression [32]. In the same study, the values of reliability of the FertiQoL scale were considered high, ranging from 0.72 to 0.91 in couples who received assisted reproductive technology (ART) and non-ART treatment [32].

Based on the CFA and in terms of the number of domains loading on each of the treatment and core subscales, the current study revealed that these two subscales explained 48% of the shared covariance between the analyzed indicators of quality of life. The two subscales were also found to be significantly and positively correlated with each other, denoting their convergent validity of the Arabic version of the FertiQoL. However, in the study of Maroufizadeh and colleagues [27], the CFA supported a four-factor model of the core FertiQoL subscale and a two-factor model of the treatment subscale. Likewise, the study of Priangga and colleagues [28] supported a four-factor model of the core FertiQoL subscale and a two-factor model of the treatment subscale. However, the study of Sexty and colleagues [32] reported that their CFA results showed some unsatisfactory indices, particularly with regards to the family and friends’ support items being weakly loaded on the social domain of the FertiQoL. Meanwhile, in the same study, the emotional and mind/body domains were strongly and positively intercorrelated. The study supported the original four-factor model for both female and male participants [33].

In a Korean study, the mind/body (*r* = –0.495) and emotional (*r* = –0.495) domains had significant negative correlations with stress, which supported the reliability of the FertiQoL subscales [34]. Similarly, the same study revealed significant negative correlations between each of the social (*r* = –0.537) and relational (*r* = –0.385) domains and depression [33]. In an Italian study, the CFA confirmed the four-factor model of FertiQoL with a good fit. In the same study, the CFA confirmed a higher reliability value of the FertiQoL for women than for men [24].

As for the goodness-of-fit statistics, the chi-squared (χ2) test and other indices of the goodness-of-fit test indicated an acceptable fit between the proposed model of the current study with the covariance matrix of the six subscales of the Arabic version of the FertiQoL. The results were as follows: χ2 (8) = 7.943, CMIN/DF = 0.993 (*p*-value = 0.44), TLI = 0.989, and CFI = 0.999, with RMSEA fit index = 0.001, 90% C. I (0.002, 0.083)). The values of CFI and TLI remained high, hence indicating an acceptable fit. In a previous Japanese study, reliability measured by Cronbach’s alpha (0.92) and CFA supported the six-domain model with 34 items, showing acceptable goodness-of-fit indices as follows: adjusted goodness of fit index = 0.855, CFI = 0.893, and RMSEA = 0.059 [34].

## Limitations

Although this study is the first to assess the psychometric properties of FertiQoL in Jordanian infertile couples, some limitations may limit the generalizability of the results. First, participants were only recruited from the northern region of Jordan, which may not represent all infertile couples in Jordan. Second, the use of a self-administered questionnaire may have affected the credibility of the responses, as some participants may have been reluctant to disclose their actual responses and therefore may not have answered truthfully some sensitive questions.

## Conclusion

The Arabic version of FertiQoL is reliable and valid for use with Jordanian infertile couples. The two FertiQoL subscales explained 48% of the common covariance between the analyzed quality of life indicators. The indices of goodness-of-fit of the model examined in the current study showed an acceptable fit mode between the six subscales of the Arabic version of FertiQoL the two latent quality of life domains (core and treatment) were significantly and positively correlated with each other, indicating their convergent validity. Therefore, the Arabic version of FertiQoL is a valid and reliable tool and can be used by healthcare providers, including nurses, to assess the emotional, physical, relational, and social quality of life challenges of Jordanian infertile couples. Furthermore, the study findings can be used to carry out longitudinal research studies to evaluate and monitor how couples deal with the psychological and physiological effects of infertility on their quality of life throughout their journey. As well as, conducting cross-cultural comparisons of factors affecting infertile couples’ quality of life using FertiQoL as an international infertility-specific measurement tool. Consequently, intervention measures and counseling programs can be developed and tailored to meet their health needs.

## Data Availability

The datasets generated during and/or analyzed during the current study are available from the corresponding author on reasonable request.
